# Reuse of Food Waste: The Chemical Composition and Health Properties of Pomelo (*Citrus maxima*) Cultivar Essential Oils †

**DOI:** 10.3390/molecules27103273

**Published:** 2022-05-19

**Authors:** Natale Badalamenti, Maurizio Bruno, Rosario Schicchi, Anna Geraci, Mariarosaria Leporini, Rosa Tundis, Monica Rosa Loizzo

**Affiliations:** 1 Department of Biological, Chemical and Pharmaceutical Sciences and Technologies (STEBICEF), University of Palermo, 90128 Palermo, Italy; natale.badalamenti@unipa.it (N.B.); maurizio.bruno@unipa.it (M.B.); anna.geraci@unipa.it (A.G.); 2 Centro Interdipartimentale di Ricerca, Riutilizzo Bio-Based Degli Scarti da Matrici Agroalimentari (RIVIVE), University of Palermo, 90128 Palermo, Italy; 3 Department of Agricultural, Food and Forest Sciences (SAAF), University of Palermo, 90128 Palermo, Italy; rosario.schicchi@unipa.it; 4 Department of Pharmacy, Health and Nutritional Sciences, University of Calabria, 87036 Rende, Italy; marilepo87@gmail.com (M.L.); monica_rosa.loizzo@unical.it (M.R.L.)

**Keywords:** *Citrus maxima* (Burm.) Merr., gas chromatography-mass spectrometry, monoterpene hydrocarbons, ‘recirculate’, PCA and HCA analyses, carbohydrate-hydrolysing enzymes, lipase

## Abstract

The aim of the present study is to investigate the chemical profile, antioxidant activity, carbohydrate-hydrolysing enzyme inhibition, and hypolipidemic effect of essential oils (EOs) extracted from Sicilian Citrus maxima (pomelo) flavedo. Using gas-chromatography-mass spectrometry analysis (GC-MS) we analysed the Eos of five cultivars of C. maxima, namely, ‘Chadock’, ‘Maxima’, ‘Pyriformis’, ‘Terracciani’, and ‘Todarii’, and their blends. The antioxidant activity was performed by using a multi-target approach using 2,2′-Azino-Bis-3-Ethylbenzothiazoline-6-Sulfonic acid (ABTS), 2,2-Diphenyl-1-picrylhydrazyl (DPPH), ferric reducing ability power (FRAP), and β-carotene bleaching tests. The α-amylase, α-glucosidase, and lipase-inhibitory activities were also assessed. GC-MS analyses revealed D-limonene as the main monoterpene hydrocarbon in all cultivars, albeit with different percentages in the range of 21.72–71.13%. A good content of oxygenated monoterpenes was detected for all cultivars, especially for ‘Todarii’. The analysis of the principal components (PCA), and related clusters (HCA), was performed to find chemo-diversity among the analysed samples. EOs from ‘Chadock’ and ‘Maxima’ were statistically similar to each other, and they differed from P3 in the smaller amount of sesquiterpene hydrocarbons, while the oils from ‘Terracciani’ and ‘Todarii’ were found to be chemically and statistically different. ‘Chadock’ EO was the most active to scavenge radicals (IC_50_ values of 22.24 and 27.23 µg/mL in ABTS and DPPH tests, respectively). ‘Terracciani’ EO was the most active against both lipase and α-amylase, whereas the blends obtained by the combination (1:1 *v*/*v*) of C. maxima ‘Maxima’ + ‘Todarii’ were the most active against α-glucosidase. Generally, the blends did not exert a unique behaviour in potentiating or reducing the bioactivity of the pomelo EOs.

## 1. Introduction

Some of the key words in the field of Green Chemistry are ‘recirculation, reuse’, aimed at highlighting environmental sustainability through the use, or rather the reuse, of food waste, chemical by-products, and refuse as result of industrial processes. However, it is often not that easy to transform, efficiently and profitably, waste into a resource. To a great extent, chemistry intervenes, as much as possible, in the food sector to recover reusable by-products. *Citrus* fruits, for example, together with the production of apples and bananas, represent the largest production of fruit cultivation in the world [1,2,3], involving continents, such as Asia, Europe, and South America [4]. They are the main fruits consumed during the wintertime in a Mediterranean diet, representing one of the most important sources of phytochemicals in the population [5]. Despite the extensive production of juices, about 50% of the fruit’s mass remains unusable for food purposes. In fact, it is estimated that *Citrus* waste (seeds and peels) produces an amount equal to 48 million tons in the world. In recent decades, several research groups [6,7,8], engaging ideas and research, have thoroughly investigated the reuse of *Citrus* peels from an environmental and sustainable perspective. In most cases, the peels are landfilled, incinerated, or composted; in some countries, on the other hand, they are used to enrich animal feed [6]. However, its use as humus is problematic: the low nitrogen content and the highly acidic pH value prevent its rapid decomposition [4], to the detriment of soil micro-organisms for their antimicrobial properties [8]. However, for a circular biochemical perspective, the possibility to extract compounds of high biological value [9], such as the antimicrobial D-limonene [10], lead to a more profitable exploitation of these wastes. The Italian *Citrus* supply is localised to the southern regions of the country, with Sicily and Calabria together producing more than 80% of the total production. Oranges represent more than 60% of the total supply, followed by clementines (17%), lemons (16%), mandarins (5%), grapefruits, and other citrus fruits for the residual part [11].

Sicily is one of the most relevant biodiversity hotspots in the Mediterranean area, with a vascular flora of 3252 species and 321 exclusive endemic taxa. The traditional Sicilian agroecosystems, influenced by territorial and economic aspects, are a heterogeneous mosaic rich in varied characteristics, connecting the ecological aspects and supporting a high percentage of rare species or species of interest for conservation, such as the genus *Citrus*, widely cultivated in all regions [12]. In Sicily, *Citrus* fruits are consumed fresh or processed in juices and candied fruit [11].

The principal wastes deriving from *Citrus* processing are flavedo and seeds. Several pieces of research have proved that these matrixes are still rich in a bioactive class of compounds, such as terpenes, flavonoids, and limonoids [13,14,15,16,17,18,19,20]. Moreover, essential oils (EOs) should be obtained from flavedo. These EOs can be applied for their pleasant aroma to both food and cosmetic products [11].

In the food industry, *Citrus* EOs are used to protect the food matrix from rancidity and/or loss of nutritional quality, colour and flavour, and microbial contamination [20].

Among *Citrus* fruits, pummelo (*Citrus maxima* (Burm.) Merr.), native to Southeast Asia, is one of the true species of *Citrus*, along with *C. reticulata* Blanco and *C. medica* L., based on both and analysis of biochemical polymorphism [21,22] and investigations on the karyotype [23]. The idea that these three species are the ancestors of cultivated taxa, is supported by further analyses using different molecular markers [24,25,26,27,28,29,30].

The pummelo, which shows a high morphological variability on the characters of the fruit, such as shape (oblate spheroid or sub-pyriform), size, thickness of the peel, colour of the pulp, and flavour, produces the largest fruits in *Citrus* species, reaching up to 3 kg in weight per fruit [31]. Pomelo EO, obtained from peels, has a characteristic odour, and it has numerous applications in fragrant, aromatherapeutic, spiritual, and cosmetic fields [32]. Volatiles of *C. maxima* EOs are mainly composed of mono- and sesquiterpene hydrocarbons and their oxygenated derivatives as well as linear hydrocarbons, alcohols, aldehydes, and esters [33,34].

**Figure 1:**
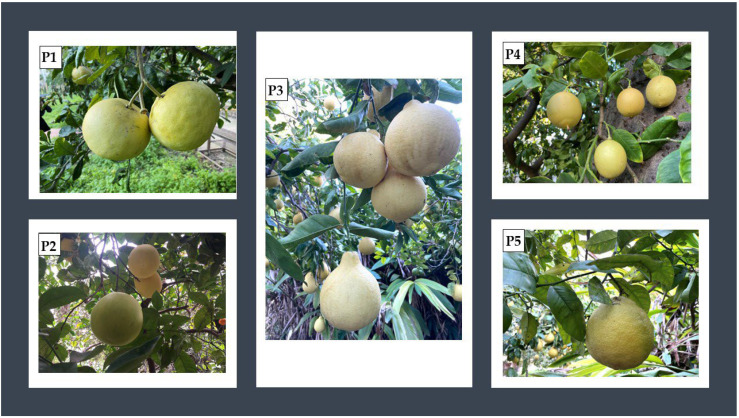
The five *C. maxima* cultivars grown in the Palermo Botanical Garden: *C. maxima* ‘Chadock’ (**P1**), *C. maxima* ‘Maxima’ (**P2**), *C. maxima* ‘Pyriformis’ (**P3**), *C. maxima* ‘Terracciani’ (**P4**), and *C. maxima* ‘Todarii’ (**P5**).

In recent years, the prevalence of obesity has increased worldwide. This situation turns out to be a major public health problem since these conditions are associated with a risk of morbidity and death from conditions, including metabolic diseases [35]. Obesity is not just the case of being overweight, but a metabolic disorder due to the accumulation of excess dietary calories into visceral fat and the release of high concentrations of free fatty acids into various organs. Recent studies may indicate that excess body fat is enough to increase oxidative stress, which suggests that free radicals can play an important role in the aetiology and development of comorbidity related to obesity, such as hyperglycaemia, leading to type 2 diabetes [36]. Hence, there is the need to identify the functional ingredients from the dual activity. In our continuous search of the potential reuse of *Citrus*-industry waste, we investigate, in the present study, the chemical composition of the EOs of five different cultivars of pomelo (*C. maxima*), ‘Chadock’ (P1), ‘Maxima’ (P2), ‘Pyriformis’ (P3), ‘Terracciani’ (P4), and ‘Todarii’ (P5) (Figure 1), cultivated in Palermo, Sicily alone and in combination (1:1 *v*/*v*) to assess the potential effect of the synergism or antagonism of action. Moreover, pomelo EOs have been evaluated for their antioxidant potential using different in vitro assays (ABTS, DPPH, FRAP, and the *β*-carotene bleaching test).

The α-amylase-, α-glucosidase-, and lipase-inhibitory activities were also performed. The inhibition of α-amylase and α-glucosidase, enzymes that are involved in the digestion of carbohydrates, can considerably reduce the post-prandial increase in blood glucose, and can therefore be a good strategy for the management of blood glucose levels in type 2 diabetic and borderline patients. Instead, the inhibition of pancreatic lipase is the most widely investigated mechanism for the identification of potential anti-obesity agents.

## 2. Results and Discussions

### 2.1. Composition of EOs and Their Blends

Flavedo hydro-distillation produced yellow oils. Overall, forty-two compounds were identified and listed in Table 1 according to their retention indices on a DB-5MS non-polar column and classified into five classes: monoterpene hydrocarbons (MHs), oxygenated monoterpenes (OMs), sesquiterpene hydrocarbons (SHs), oxygenated sesquiterpenes (OSs), and other compounds (Os).

From an examination of the results, a possible division of the cultivars into three different classes emerged: a group, constituted of *C. maxima* P1, P2, and P3 EOs, rich in monoterpene hydrocarbons (83.47–85.41%), dominated by the clear presence of D-limonene (58.66–71.13%), and a moderate quantity of oxygenated ones (11.01–14.72%) caused by the presence of compounds, such as *cis*-linalool oxide, β-linalool, α-terpineol, and α-citral. The P4sample, compared to the group described above, had a smaller quantity of monoterpene hydrocarbons (49.92%) and a greater quantity of oxygenated monoterpenes (26.27%).

This cultivar is always characterised by the clear presence of D-limonene (32.42%), but also by good quantities of β-pinene (13.99%) and α-citral (8.50%). Furthermore, there was the presence of OS (nootkatone: 4.45%) and compounds belonging to the chemical class (O), especially alcohol (1-octanol and 1-nonanol) and aldehydes (octanal, nonanal, and decanal).

The *C. maxima* ‘Todarii’ cultivar (P5) differed greatly from the other cultivars. In fact, it was characterised by the total absence of sesquiterpenes (SHs and OSs), and other compounds and, from the GC-MS analysis, the quantity of hydrocarbon and oxygenated monoterpenes was practically comparable (45.91 and 44.41, respectively). D-Limonene (21.72%) and β-linalool (19.58%) were the majority volatiles, but moderate amounts of 4-carene (7.90%), *α*-terpineol (6.20%), neryl acetate (4.64%), geranyl acetate (4.70%), and *γ*-terpinene (4.57%) were recorded.

Table 2 reports the chemical variation of the blends obtained by combining equi-volumetric quantities of the individual EOs. The different samples, from P1P2 to P4P5, were obtained by mixing two oils in equal parts; only the sample ‘mix’ (P1 + P2 + P3 + P4 + P5) was obtained for equal miscibility of the EOs extracted from the five cultivars. GC-MS analysis confirmed for almost all the samples, except for P4P5, the strong presence of compounds belonging to the class of monoterpene hydrocarbons (64.74–84.65%), with a high percentage of D-limonene (40.20–66.93%) and a modest amount of β-pinene (2.75–11.90%) and β-myrcene (2.66–5.65%). Furthermore, all these samples were also characterised by the relative presence of oxygenated monoterpenes (10.76–29.53%), such as β-linalool, α-terpineol, and two isomers, neryl acetate and geranyl acetate, and very low quantities of sesquiterpene compounds (0.18–2.94%).

The P4P5 sample, on the other hand, had a chemical composition dictated by both oxygenated and hydrocarbon monoterpenes (35.36–47.95%, respectively). The amount of D-limonene was significantly lower than in all other mixes (27.08%), and greater amounts of β-linalool (11.43%), *α*-terpineol (4.03%), *cis* and *trans*-geraniol (3.38 and 3.88%, respectively), and α-citral (4.25%) were recorded.

The research, conducted using the major scientific systems (Scopus, SciFinder, Google Scholar), highlighted the absence of information on the composition and/or on the possible biological applications of EOs for the varieties ‘Terracciani’, ‘Chadock’, ‘Pyriformis’, and ‘Todarii’. Instead, several scientific works reported the chemical composition of the EOs produced from the flavedo of C. maxima: the analysis conducted by Tao and Liu [37] showed, for this cultivar, an essential oil practically made up of D-limonene (89.96% ) and 4.46% of β-myrcene, but with very low abundances of SH and OS; pomelo of C. maxima, grown in Vietnam, confirmed the high presence of hydrocarbon monoterpenes, D-limonene among all, and also, in this case, small percentages of sesquiterpene compounds [38]. This confirmed the results of the performed analyses, finding both a high percentage of MH and low percentage (<1%) of SH and OS. The difference compared to our oil is the higher presence of OM.

### 2.2. PCA and HCA Analyses of the EOs and Their Mixes

The statistical analyses were conducted considering the different chemical classes in which the different chemical compounds identified on the GC-MS were releveled, according to the loading plot obtained by principal component analysis (PCA) for monoterpene hydrocarbons (MH), oxygenated monoterpenes (OM), sesquiterpene hydrocarbons (SH), oxygenated sesquiterpenes (OS), and other compounds (O).

**Figure 2:**
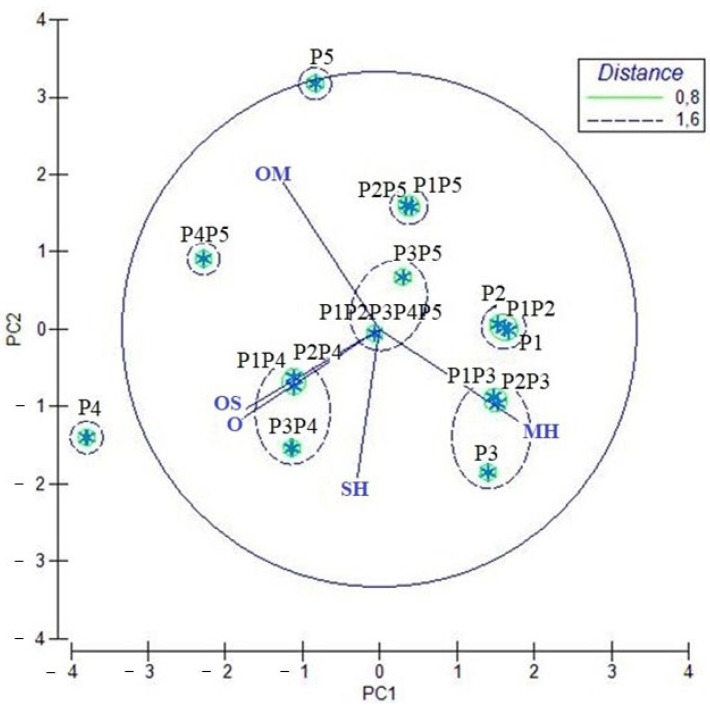
Principal component analysis (PCA) of EOs from C. maxima and their mixes based on the principal classes of compounds: monoterpene hydrocarbons (MH), oxygenated monoterpenes (OM), sesquiterpenes hydrocarbons (SH), and oxygenated sesquiterpenes (OS), and others (O). The vectors shown are the eigenvectors of the covariance matrix. The samples’ codes are reported in Section 2.1: Plant Material.

For the EOs of the different C. maxima cultivars and their blends, as shown in the loading graph (Figure 2), all variables influenced PC1 and PC2. The PCA of C. maxima EO presented a total variance of 87.2% of the original data. In fact, PC1 (51.5% of the total significant contribution) was mainly represented by oxygenated monoterpenes and sesquiterpenes (OMs and OSs, respectively), and by other compounds (O), in the negative score, by an MH eigenvalue with a positive score, while the SH was statistically irrelevant; meanwhile, PC2 (35.7%) was represented by a positive score of OM, but to a greater level by MH, SH, OS, and O compounds.

**Figure 3:**
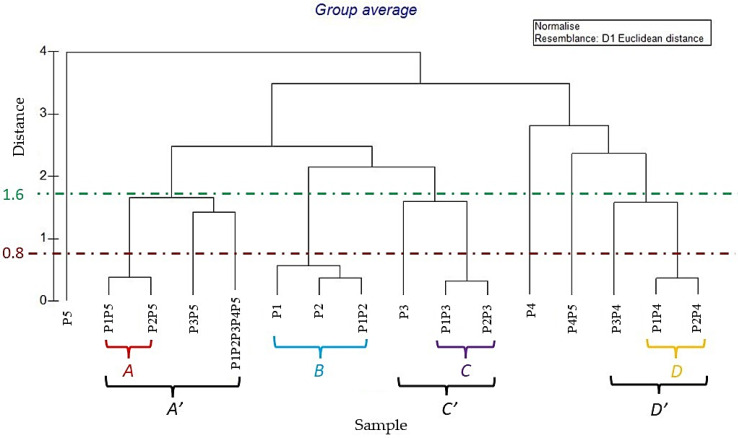
Dendrogram obtained by Hierarchical Cluster Analysis (HCA) based on the Euclidian distances. The samples’ codes are reported in Section 2.1: Plant Material.

HCA based on the Euclidean distance between groups indicated a solution with four clusters (A, B, C, and D), with a distance < 0.8 (Figure 3), which was mainly due to the variation along the major axis in the PCA analysis. These clusters formed separate groups in the PCA biplot (Figure 2).

With a dissimilarity < 0.8 (red line), four clusters existed. The first one, group A, was represented by P1P5 and P2P5 EOs mixes, characterised by a high percentage of MH (65.62 and 64.74%, respectively) and a moderate content of OM (27.50 and 29.53%). Group B included samples P1, P2, and P1P2, represented by an identical quantity of compounds divisible into the two monoterpene classes and slightly different for the percentages of sesquiterpenes. Mixes P1P3 and P2P3 formed the cluster C: both mixtures had identical quantities of all the chemical classes taken into consideration, even of the compounds belonging to the O class (0.55 and 0.50%, respectively). Finally, group D consisted of two samples, P1P4 and P2P4, rich in MH compounds, but unlike the other clusters, they presented not insignificant quantities of SO (2.94–2.64%, respectively) and O compounds (4.10–4.02%).

Increasing the level of dissimilarity (cut-off = 1.6, Figure 3, green line), the macro-clusters became three. Cluster A’, which, in addition to the inclusion of cluster A, also incorporated samples P3P5 and P1 + P2 + P3 + P4 + P5, which was slightly different in comparison to P1P5 and P2P5, in the content of SH and O constituents. Sample P3 could be contained within the C cluster, forming the C’ group, although it did not have oxygenated sesquiterpenes and had a higher percentage of other compounds. Finally, cluster D was enlarged to D’ by incorporating the P3P4 sample not previously included due to the slightly higher content of SH compounds.

The samples P4, P5, and P4P5, due to the dissimilar percentages of OM and O compared to the other mixes, could not be included in any possible cluster. As is already evident from Table 1 and Table 2, their composition was distinctly different from all other EOs and related blends, and the statistical analysis confirmed this evidence.

### 2.3. Radical Scavenging and Antioxidant Activities of Pomelo EOs and Their Blends

**Figure 4:**
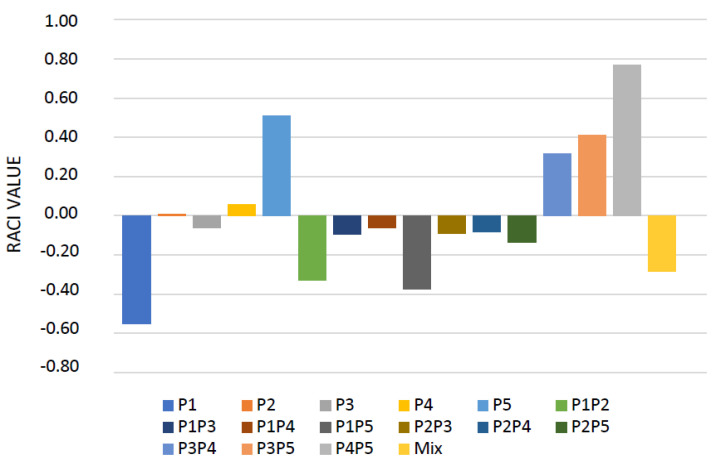
Relative Antioxidant Capacity Index by pomelo EOs and their blends. Mix: sample obtained by combination P1, P2, P3, P4, and P5 EOs (1:1 *v*/*v*).

Different in vitro assays were performed to evaluate, with a multi-target approach, the antioxidant potential of pomelo EOs and their blends obtained by the flavedo of different cultivars of C. maxima. The concentration-dependent effects were observed for all investigated samples, except for the FRAP assay (Table 3). The P1 EO was the most active in both tests applied to verify the radical scavenging potential of samples with IC_50_ values of 22.24 and 27.23 µg/mL for the ABTS and DPPH assays, respectively. A slightly lower radical scavenging potential was observed when the EOs were combined with IC_50_ values from 24.28 to 35.27 µg/mL for P1 + P2 + P3 + P4 + P5 and P4P5, respectively, in the ABTS test, and from 26.82 to 35.24 µg/mL for P1 + P2 + P3 + P4 + P5 and P2P3, respectively, in the DPPH test. It is interesting to note that, except for the blend obtained by mixing equal volumes of P2 and P3, all blends had better FRAP values than those obtained with BHT used as a positive control. A great variability was observed in the protection from lipid peroxidation. In this assay, P4 was the most active, with an IC_50_ value of 25.56 µg/mL. Among the blends, the EOs with the greatest protective power for lipid peroxidation were P1P2 and P1P3 (IC_50_ values of 26.28 and 21.87 µg/mL, respectively). Based on the Relative Antioxidant Capacity Index (RACI), which creates a ranking clustering of the antioxidant capacity for different samples, P1 showed the highest antioxidant potential with an RACI value of −0.55, followed by the P1P5 blend (RACI value of −0.38) (Figure 4).

Our results are better than those reported by Lan-Phi et al. [39], which evaluated the DPPH radical scavenging activity of different pomelo varieties, namely, DaXanh, DuongCam, Nam Roi, and Buoi Long, and found IC_50_ values in the range of 43.8–63.1 mg/mL. On the contrary, a better radical scavenging activity was observed for the Indian C. maxima EO with an IC_50_ value of 8.84 µg/mL [40], and also for pomelo from Malaysia [14].

A comparison between the antioxidant activity of pomelo distilled essential oil (DEO) and cold-pressed essential oil (CPEO) revealed that CPEO was more potent than DEO in the total reducing power and radical scavenging activity [41].

### 2.4. Inhibition of Enzymes Involved in Metabolic Syndrome and Obesity by Pomelo EOs and Their Blends

The inhibition of lipase, as well as carbohydrate-hydrolysing enzymes, α-glucosidase, and α-amylase, was evident in a concentration-dependent manner (Table 4). ‘Terracciani’ and ‘Todarii’ EOs exhibited the highest lipase-inhibitory activity with IC_50_ values of 23.22 and 24.23 μg/mL, respectively. The combinations of the EOs (1:1 *v*/*v*) did not significantly improve the lipase-inhibitory activity (IC_50_ values from 25.22 to 39.12 μg/mL for P2P4 for P1P4, respectively).

Regarding the carbohydrate-hydrolysing enzymes, the P4 EO was the most active against α-amylase (IC_50_ value of 25.23 μg/mL), whereas P2 was the most active against α-glucosidase (IC_50_ value of 25.67 μg/mL). Regarding blends data, the P1P4 sample was the most active against α-amylase (IC_50_ value of 21.43 μg/mL). A combination of the EOs increased the α-glucosidase-inhibitory activity, where the blend obtained by mixing equal volumes of ‘Maxima’ (P2) and Todarii’ (P5) was the most active with an IC_50_ value of 21.67 μg/mL, followed by the P1P2 blend. Our results are in agreement with those reported for the lemon flavedo EO by Oboh et al. [42], which obtained IC_50_ values of 8.16 and 7.56 μg/mL against α-amylase and α-glucosidase, respectively. A promising activity was also observed for the EO obtained by orange flavedo (IC_50_ values of 11.51 and 11.53 μg/mL, respectively).

Recently, Itoh et al. [43] analysed different Citrus by-products as possible lipase inhibitors and found IC_50_ values of 43 and 44 μg/mL for leaves and flower methanol extract, respectively. Values in the range of 135.51–282.65 μg/mL were obtained for EOs obtained from *C. × clementina* leaves collected in Rosarno and Cetraro, respectively, against α-amylase and α-glucosidase, whereas values in the range of 185.43–287.91 μg/mL were obtained for EOs obtained from flavedo of fruits of the same species collected in Corigliano Calabro and Rosarno, respectively [44,45]. The EO from *C. × clementina* flavedo exhibited lipase-inhibitory activity, although with lower potency than the pomelo EOs. [44]. Dang [46] did not obtain a-glucosidase-inhibitory activity for pomelo EO from Vietnam. According to Dang et al. [46], D-limonene was the main abundant compound in pomelo oils; although this is presented in the literature as an antidiabetic phytochemical, it is not responsible of the carbohydrate-hydrolysing enzyme-inhibitory activity [47,48]. On the contrary, α-pinene, β-pinene 1,8-cineole, 4-terpineol, and α-terpineol resulted in being able to inhibit α-amylase [49], and the activity of the EO is often the result of an antagonistic or additive effect between the constituents of the oil.

## 3. Materials and Methods

### 3.1. Plant Material

The five *C. maxima* samples analysed, cultivated in the Botanical Garden of Palermo (38°06′48.39″ N; 13°22′21.68″ E), Sicily, were collected in February 2020. In the present work, fruits belonging to the following five cultivars were examined: *C. maxima* ‘Chadock’ (P1), *C. maxima* ‘Maxima’ (P2), *C. maxima* ‘Pyriformis’ (P3), *C. maxima* ‘Terracciani’ (P4), and *C. maxima* ‘Todarii’ (P5). The samples, identified by Prof. Rosario Schicchi and Prof. Anna Geraci, were kept in the “Herbarium Mediterraneum” of the Botanical Garden of the University of Palermo (PAL). The number of the voucher was reported for each cultivar. *C. maxima* ‘Chadock’ (P1) (Voucher No. 109746) is characterised by large, flattened, turbinate fruits, which are slightly umbilicate at the apex. The leaves are ovate-oblong, with a winged petiole, especially for those at the ends of twigs. *C. maxima* ‘Maxima’ (P2) (Voucher No. 109743) is characterised by very large fruits, cylindrical in shape, and slightly narrowed at the insertion of the peduncle. The leaves are ovate-oblong, quite curled, with a slightly winged petiole. *C. maxima* ‘Pyriformis’ (P3) (Voucher No. 109742) is characterised by small–medium-sized pyriform fruits, tapering at the insertion of the peduncle, and with a small protuberance at the apex. The leaves are ovate-oblong with a slightly winged petiole. *C. maxima* ‘Terracciani’ (P4) (Voucher No. 109744) is characterised by small, oval-shaped fruits with a persistent style and stigma. The leaves are ovate-oblong, with a slightly winged petiole. *C. maxima* ‘Todarii’ (P5) (Voucher No. 109745) is characterised by medium–large, mostly globular, fruits with a slightly depressed base. The leaves are ovate-oblong, with a slightly winged petiole.

### 3.2. Essential Oil Extractions and Their Blend Preparation

The extraction of EOs was performed according to Basile et al. [50]. Variable quantities of flavedos of P1, P2, P3, P4, and P5 (47–121 g) were subjected to hydro-distillation for 3 h using Clevenger’s apparatus [51] to obtain the oils with yields of 0.73%, 1.03%, 0.62%, 0.35%, and 0.47% (v/w), respectively. A combination of EOs was obtained by mixing oils in a 1:1 *v/v* ratio. EOs and their blends were stored in the freezer at −20 °C, until the time of analysis.

### 3.3. Pomelo EOs' Volatile Profiles

The analyses of EOs and their blends were performed according to the procedure reported by Catinella et al. [52]. Chromatograms of all EOs are reported in Supplementary Materials (Figures S1–S5). Linear retention indices (LRIs) were determined by using the retention times of *n*-alkanes (C_8_–C_40_), and the peaks were identified by comparison with mass spectra and by comparison to their relative retention indices with WILEY275 (Wiley), NIST 17 (NIST, The National Institute of Standards and Technology, Gaithersburg, MD, USA), ADAMS [53], and FFNSC2 (Shimadzu, Kyoto, Japan) libraries.

### 3.4. Evaluation of Radical Scavenging Activity by ABTS and DPPH Assays

Pomelo EOs and their combinations were studied for their radical scavenging activity by using 2,2’-azino-bis-3-ethylbenzthiazoline-6-sulphonic acid (ABTS) and 1,1-diphenyl-2-picrylhydrazyl (DPPH) tests [13].

Briefly, a solution of ABTS^+^ radical, opportunely prepared, was diluted with EtOH to reach an absorbance of 0.70 at 734 nm. EOs were added to a diluted ABTS^+^ solution to test the concentration from 400 to 1 μg/mL and, following incubation, the absorbance was read at 734 nm. The procedure to test the DPPH radical scavenging potential of samples was assessed, as previously described [13]. Briefly, the EOs (1–1000 μg/mL) were mixed with DPPH· radical solution and, following the incubation time, the absorbance was read at 517 nm. Ascorbic acid was used as a positive control in both tests.

### 3.5. Ferric Reducing Ability Power (FRAP Assay)

The Ferric Reducing Ability Power (FRAP) test was used, as previously reported [13]. The EOs (100 μL) were mixed with water and FRAP reagent. After 30 min of incubation, the absorbance was read at 595 nm. The FRAP value was expressed as μM Fe(II)/g. Butylated hydroxytoluene (BHT) was used as a positive control.

### 3.6. β-Carotene Bleaching Test

The protection from lipid peroxidation by pomelo EOs and their combination was assessed using the β-carotene bleaching test [13]. Briefly, β-carotene, Tween 20, and linoleic acid were mixed. The resulting emulsion was added to a 96-well microplate containing EOs in concentrations ranging from 100 to 2.5 μg/mL. Following incubation, the absorbance was read at 470 nm. Propyl gallate was used as a positive control.

### 3.7. Lipase-Inhibitory Assay

To assess the potential anti-obesity effects of pomelo EOs and their mixes, the activity of porcine pancreatic lipase (type II) (EC 3.1.1.3) activity was measured using *p*-nitrophenyl octanoate as a substrate [54]. The EOs were mixed with the enzyme solution (1 mg/mL in water), 0.005 M of *p*-nitrophenyl octanoate solution, and Tris-HCl buffer (pH = 8.5). Following incubation at room temperature, the absorbance was read at 412 nm. Orlistat was used as a positive control.

### 3.8. Carbohydrate-Hydrolysing Enzyme-Inhibition Assay

Both α-amylase and α-glucosidase are involved in carbohydrate digestion and have been recognised as targets for postprandial hyperglycaemia modulation [55]. For the α-amylase-inhibitory assay, a starch solution of α-amylase enzyme (EC 3.2.1.1) and colorimetric reagent were prepared [54]. Pomelo oils were added to the starch solution and left to react with the enzyme. The absorbance was read at 540 nm. In the α-glucosidase-inhibitory-activity assay, α-glucosidase (EC 3.2.1.20) was mixed with a maltose solution and O-dianisidine [54]. The pomelo oils were added at different concentrations and were left to incubate at 37 °C for 30 min. Subsequently, perchloric acid was added and the mixture was centrifuged. The supernatant was collected and mixed with DIAN, PGO, and left to incubate at 37 °C for 30 min. Then, the absorbance was read at 500 nm.

### 3.9. Statistical Analysis

All analyses were performed in triplicate. Data are expressed as the means ± standard deviation (S.D.). The concentration–response curve was obtained by plotting the percentage inhibition versus concentration. The concentration that yielded 50% inhibition (IC_50_) was calculated by nonlinear regression, with the use of GraphPad Prism version 4.0 for Windows (GraphPad Software, San Diego, CA, USA). Differences within and between the groups for antioxidant assays were evaluated by one-way ANOVA followed Tukey’s test, and was applied in order to determine any significant differences among the investigated samples (** *p* < 0.05). To obtain a ranking of the EOs and their mixes, the antioxidant capacity Relative Antioxidant Capacity Index (RACI) was calculated following the procedure described by Todorovic et al. [56]. This statistical application was generated from the perspective of statistics by integrating the antioxidant-capacity values generated from different in vitro methods.

Principal component analysis was performed according to the procedure reported by Badalamenti et al. [13]. The different chemical classes used to describe the composition of individual essential oils and their mixes were considered as original variables and subjected, after normalisation, to cluster analysis (CA) and to principal component analysis (PCA). The statistical analyses were performed using PRIMER 6 (Massey University Eastbourne, Albany, New Zealand) with two principal component (PC) variables, and the number of clusters were determined by using the rescaled distances in the dendrogram, using a cut-off point (Euclidean distance = 0.8) that allowed for the attainment of consistent clusters. The Principal Components Analysis (PCA) and the Hierarchical Cluster Analysis (HCA) were used to comprehend the similarity among the essential oils in relation to the contents of their chemical constituents. We tested two different cut-off similarity levels (cut-off levels 0.8 and 1.6) that were chosen based on the mean distance between the cluster’s measure and the similarities–differences between the samples belonging to the same cluster. Since the HCA analysis is a function of variables and observations, the highest correspondence between PCA and HCA was produced when we applied a cut-off of 0.8. The statistical analysis of the absence/presence was conducted using the cluster method of the PRIMER 6 software (Massey University Eastbourne, Albany, New Zealand).

## 4. Conclusions

EOs obtained by the flavedo of C. maxima cultivars ‘Chadock’, ‘Maxima’, ‘Pyriformis’, ‘Terracciani’, and ‘Todarii’, collected from the Palermo Botanical Garden, were chemically and biologically investigated, alone and as combinations. D-Limonene was the main abundant monoterpene hydrocarbon in all cases. Chemical and statistical analyses (PCA and HCA) can provide chemodiversity information on the investigated samples. A division of the cultivars into four classes emerged: the first one, composed of P1P5 and P2P5 samples, was characterised by a high percentage of MH and a moderate content of OM. Group B included samples characterised by an equal content of monoterpenic compounds. The P1P3 and P2P3 mixes, chemically similar in all classes, formed the third group. The last group, cluster D, was, instead, made up of samples with the same content of monoterpenic compounds, oxygenated sesquiterpenes, and other metabolites. On the other hand, more detailed PCA and HCA analyses showed the clustering of EOs into three groups: cluster A’ can incorporate samples P3P5 and P1P2P3P4P5, which were slightly different, compared to group A, in the content of SH and O constituents. Cluster C’ was formed by inclusion in group C of sample P3, containing a higher percentage of other compounds. Finally, cluster D was enlarged to D’ by incorporating the P3P4 sample, not previously included due to the slightly higher content of SH compounds.

All tested Sicilian Pomelo EOs and their blends exhibited a promising antioxidant activity trough different mechanisms of action. Among them, pomelo from ‘Chadock’ was the most active, followed by the blend obtained by an equal-volume combination of ‘Chadock’ and ‘Todarii’ cultivars. Regarding the inhibitory activity against the enzymes involved in metabolic syndrome, C. maxima ‘Terracciani’ and ‘Todarii’ EOs exhibited the highest lipase-inhibitory activity. Moreover, all EOs were able to inhibit carbohydrate-hydrolysing enzymes without any significant differences between the EO tested alone and as a blend.

According to our previous data, the bioactivity was not strictly related to the main compound: D-limonene. This was probably due to the common effect observed for natural products when “1 + 1 does not equal 2” [57]. In fact, minor compounds can significantly contribute to the functional properties of EOs, in which they sometimes act synergically.

This study evidenced, for the first time, the chemical and biological properties of Sicilian pomelo essential oils. However, further studies are necessary to identify the possible application of pomelo oils as functional ingredients for the development of functional food or nutraceutical products, alone or as a blend.

## Figures and Tables

**Table 1:** Composition (%) of EOs of the five *C. maxima* cultivars collected in Sicily.

				Content (%) ^C^						
No.	Compounds	LRI_exp_ ^A^	LRI_exp_ ^B^	P1	P2	P3	P4	P5	Ident. ^D^	Sign. ^E^
1	α-Pinene	937	1008	1.92 ± 0.19 ^d^	2.97 ± 0.25 ^c^	4.03 ± 0.53 ^a^	3.23 ± 0.36 ^b^	1.80 ± 0.14 ^e^	1, 2, 3	**
2	Camphene	948	1069	0.01 ± 0.01 ^b^	0.07 ± 0.05 ^a b^	0.19 ± 01 ^a^	-	-	1, 2, 3	**
3	β-Pinene	979	1102	4.52 ± 0.56 ^d^	8.23 ± 0.84 ^c^	9.83 ± 1.02 ^b^	13.99 ± 1.08 ^a^	1.00 ± 0.95 ^e^	1, 2, 3	**
4	β-Myrcene	988	1162	5.31 ± 0.61 ^b^	5.38 ± 0.71 ^b^	5.91 ± 0.62 ^a^	-	4.72 ± 0.37 ^c^	1, 2, 3	**
5	Octanal	1005	1265	-	-	-	3.53 ± 0.28 ^a^	-	1, 2	**
6	α-Phellandrene	1009	1178	0.08 ± 0.07 ^d^	0.72 ± 0.12 ^b^	1.55 ± 0.21 ^a^	-	0.22 ± 0.11 ^c^	1, 2	**
7	4-Carene	1013	1157	-	-	-	0.28 ± 0.05 ^b^	7.90 ± 0.89 ^a^	1, 2	**
8	β-Phellandrene	1017	1197	1.25 ± 0.21 ^c^	2.61 ± 0.18 ^b^	3.21 ± 0.32 ^a^	-	0.07 ± 0.05 ^d^	1, 2	**
9	o-Cymene	1021	1266	-	-	-	-	0.66 ± 0.09 ^a^	1,2	**
10	Limonene	1028	1206	71.13 ± 2.7 ^a^	62.73 ± 1.9 ^b^	58.66 ± 1.7 ^c^	32.42 ± 1.2 ^d^	21.72 ± 1.1 ^e^	1, 2, 3	**
11	β-cis-Ocimene	1040	1234	-	-	-	-	1.67 ± 0.25 ^a^	1, 2	**
12	γ-Terpinene	1047	1246	1.19 ± 0.21 ^b^	0.76 ± 0.02 ^c^	-	-	4.57 ± 0.52 ^a^	1, 2	**
13	1-Octanol	1071	1549	-	-	-	1.13 ± 0.09 ^a^	-	1, 2	**
14	cis-Linalool oxide	1073	1435	1.51 ± 0.21 ^b^	5.51 ± 0.61 ^a^	-	0.62 ± 0.07 ^c^	0.19 ± 0.0 ^d^	1, 2	**
15	β-Linalool	1084	1552	2.05 ± 0.36 ^c^	2.16 ± 0.11 ^c^	2.15 ± 0.18 ^c^	3.28 ± 0.24 ^b^	19.58 ± 1.3 ^a^	1, 2, 3	**
16	α-Terpinolene	1088	1287	-	-	0.52 ± 0.09 ^b^	-	0.87 ± 0.10 ^a^	1, 2	**
17	*trans*-Linalool oxide	1095	1473	0.79 ± 0.08 ^b^	2.27 ± 0.31 ^a^	-	0.34 ± 0.02 ^c^	0.13 ± 0.0 ^d^	1, 2	**
18	Heptyl acetate	1098	1377	-	-	-	0.20 ± 0.01 ^a^	-	1, 2	**
19	neo-allo-Ocimene	1136	1354	-	-	-	-	0.71 ± 0.08 ^a^	1, 2	**
20	β-Citronellal	1138	1490	-	-	-	0.73 ± 0.09 ^a^	-	1, 2	**
21	Nonanal	1143	1374	-	-	0.30 ± 0.02 ^b^	1.26 ± 0.12 ^a^	-	1, 2	**
22	*trans*-3-Pinanone	1165	1532	-	-	-	0.04 ± 0.0 ^a^	-	1, 2	**
23	1-Nonanol	1179	1645	-	-	-	1.24 ± 0.17 ^a^	-	1, 2	**
24	4-Terpineol	1184	1597	0.44 ± 0.05 ^d^	0.91 ± 0.10 ^c^	1.76 ± 0.18 ^a^	1.56 ± 0.21 ^b^	-	1, 2, 3	**
25	α-Terpineol	1196	1695	1.06 ± 0.11 ^e^	2.22 ± 0.26 ^c^	3.05 ± 0.35 ^b^	1.84 ± 0.18 ^d^	6.20 ± 0.75 ^a^	1, 2	**
26	Decanal	1205	1485	-	-	-	0.99 ± 0.10 ^a^	-	1, 2	**
27	cis-Geraniol	1228	1806	0.84 ± 0.09 ^e^	0.63 ± 0.08 ^d^	1.35 ± 0.15 ^c^	2.11 ± 0.25 ^b^	4.62 ± 0.51 ^a^	1, 2	**
28	β-Citral	1241	1678	1.75 ± 0.75 ^b^	0.36 ± 0.02 ^d^	1.20 ± 0.18 ^c^	3.68 ± 0.43 ^a^	-	1, 2	**
29	*trans*-Geraniol	1252	1843	0.49 ± 0.05 ^d^	0.66 ± 0.05 ^c^	0.44 ± 0.0 ^d^	3.57 ± 0.37 ^b^	4.19 ± 0.51 ^a^	1, 2	**
30	1-Decanol	1267	1760	-	-	0.78 ± 0.09 ^a^	-	-	1, 2	**
31	α-Citral	1272	1723	1.53 ± 0.21 ^b^	-	1.06 ± 0.18 ^c^	8.50 ± 0.76 ^a^	-	1, 2	**
32	Linalyl propionate	1314	1678	-	-	-	-	0.16 ± 0.0 ^a^	1, 2	**
33	Neryl acetate	1365	1722	-	-	-	-	4.64 ± 0.4 ^b^	1, 2	**
34	Geranyl acetate	1383	1751	-	-	-	-	4.70 ± 0.3 ^b^	1, 2	**
35	Caryophyllene	1421	1583	-	0.34 ± 0.02 ^b^	0.95 ± 1.01 ^a^	0.53 ± 0.05 ^a,b^	-	1, 2, 3	**
36	α-Bergamotene	1438	1565	-	-	-	0.79 ± 0.09 ^a^	-	1, 2	**
37	β-Bisabolene	1507	1728	-	-	1.27 ± 0.15 ^a^	-	-	1, 2	**
38	Epiglobulol	1571	2047	-	-	-	0.15 ± 0.0 ^a^	-	1, 2	**
39	*trans*-Nerolidol	1573	2053	-	0.50 ± 0.08 ^a^	-	-	-	1, 2	**
40	Nerolidyl acetate	1680	2272	-	-	-	0.08 ± 0.02 ^a^	-	1, 2	**
41	cis-Farnesol	1687	2339	0.88 ± 0.09 ^a^	-	-	-	-	1, 2	**
42	Nootkatone	1806	2505	-	-	-	4.45 ± 0.52 ^a^	-	1, 2	**
Monoterpene hydrocarbons				85.41	83.47	83.90	49.92	45.91		
Oxygenated monoterpenes				10.46	14.72	11.01	26.27	44.41		
Sesquiterpene hydrocarbons				-	0.34	2.22	1.32	-		
Oxygenated sesquiterpenes				0.88	0.50	-	4.68	-		
Others				-	-	1.08	8.35	-		
Total				96.75	99.03	98.21	90.54	98.67		

^A^ Linear retention index obtained for DB-5MS non-polar column; ^B^ linear retention index obtained for DB-Wax column; ^C^ content is the peak volume percentage of compounds in the essential oil sample; ^D^: 1 = retention index identical to bibliography; 2 = identification based on comparison of MS; 3 = retention time identical to authentic compounds. Compounds are classified in order of linear retention time of non-polar column. ^E^ Sign: Significance at ** *p* < 0.05. Results followed by different letters in the same line are significantly different (*p* < 0.05) by Tukey’s multiple-range test.

**Table 2:** Composition (%) of *C. maxima* EO blends (1:1 *v*/*v*).

Compounds	P1P2	P1P3	P1P4	P1P5	P2P3	P2P4	P2P5	P3P4	P3P5	P4P5	Mix ^	Sign.
α-Pinene	2.49 ± 0.21 ^d,e^	2.98 ± 0.30 ^b,c^	2.58 ± 0.26 ^d^	1.90 ± 0.21 ^f^	3.50 ± 0.34 ^a^	3.11 ± 0.29 ^b^	2.38 ± 0.20 ^e^	3.64 ± 0.31 ^a^	2.93 ± 0.27 ^b,c^	2.53 ± 0.23 ^d,e^	2.80 ± 0.25 ^c^	**
Camphene	0.07 ± 0.0 ^b^	0.10 ± 0.00 ^a b^	-	-	0.13 ± 0.01 ^a^	0.03 ± 0.00 ^c^	0.02 ± 0.00 ^c^	0.11 ± 0.01 ^a,b^	0.09 ± 0.00 ^a,b^	-	0.05 ± 0.00 ^c^	**
β-Pinene	6.38 ± 0.61 ^e^	7.18 ± 0.73 ^d^	9.25 ± 0.93 ^c^	2.75 ± 0.2 ^h^	9.03 ± 0.89 ^c^	11.10 ± 1.12 ^b^	4.63 ± 0.38 ^g^	11.90 ± 1.02 ^a^	5.43 ± 0.53 ^f^	7.50 ± 0.61 ^d^	7.53 ± 0.68 ^d^	**
β-Myrcene	5.35 ± 0.49 ^bc^	5.60 ± 0.52 ^a^	2.66 ± 0.24 ^f^	5.00 ± 0.46 ^c^	5.65 ± 0.51 ^a^	2.70 ± 0.23 ^f^	5.07 ± 0.46 ^c^	2.95 ± 0.21 ^e^	5.33 ± 0.47 ^b,c^	2.35 ± 0.21 ^g^	4.28 ± 0.38 ^d^	**
Octanal	-	-	1.72 ± 0.18 ^a,b^	-	-	1.67 ± 0.54 ^c^	-	1.78 ± 0.19 ^a,b^	-	1.82 ± 0.20 ^a^	0.70 ± 0.09 ^d^	**
α-Phellandrene	0.40 ± 0.03 ^d^	0.83 ± 0.07 ^b^	0.05 ± 0.00 ^f^	0.15 ± 0.00 ^e^	1.13 ± 0.09 ^a^	0.35 ± 0.02 ^d^	0.48 ± 0.01 ^c,d^	0.78 ± 0.05 ^b,c^	0.88 ± 0.09 ^b^	0.10 ± 0.02 ^e,f^	0.53 ± 0.04 ^c^	**
4-Carene	-	-	0.15 ± 0.00 ^d^	3.95 ± 0.42 ^b^	-	0.15 ± 0.000 ^d^	3.99 ± 0.41 ^a,b^	0.13 ± 0.0 ^d^	4.00 ± 0.35 ^a,b^	4.11 ± 0.43 ^a^	1.63 ± 0.14 ^c^	**
β-Phellandrene	1.93 ± 0.18 ^b,c^	2.23 ± 0.24 ^b^	0.63 ± 0.57 ^f^	0.65 ± 0.59 ^f^	2.90 ± 0.18 ^a^	1.30 ± 0.15 ^e^	1.35 ± 0.14 ^d,e^	1.60 ± 0.18 ^c^	1.65 ± 0.18 ^c^	0.03 ± 0.00 ^g^	1.43 ± 0.12 ^d^	**
o-Cymene	-	-	-	0.31 ± 0.02 ^a,b^	-	-	0.29 ± 0.02 ^b^	-	0.30 ± 0.03 ^a,b^	0.35 ± 0.03 ^a^	0.13 ± 0.01 ^c^	**
D-Limonene	66.93 ± 5.23 ^a^	64.90 ± 5.12 ^b^	51.78 ± 4.83 ^d^	46.43 ± 3.41 ^ef^	60.70 ± 5.02 ^c^	47.58 ± 3.56 ^e^	42.23 ± 3.24 ^g^	45.55 ± 3.41 ^f^	40.20 ± 3.07 ^h^	27.08 ± 1.89 ^i^	49.33 ± 3.63 ^d,e^	**
β-cis-Ocimene	-	-	-	0.81 ± 0.08 ^a^	-	-	0.83 ± 0.09 ^a^	-	0.81 ± 0.08 ^a^	0.82 ± 0.07 ^a^	0.33 ± 0.01 ^b^	**
γ-Terpinene	0.98 ± 0.11 ^d^	0.61 ± 0.05 ^e^	0.60 ± 0.06 ^e^	2.88 ± 0.24 ^a^	0.42 ± 0.03 ^f^	0.38 ± 0.02 ^f^	2.68 ± 0.31 ^a,b^	-	2.28 ± 0.26 ^b^	2.31 ± 0.29 ^b^	1.30 ± 0.14 ^c^	**
1-Octanol	-	-	0.54 ± 0.04 ^a^	-	-	0.55 ± 0.05 ^a^	-	0.54 ± 0.05 ^a^	-	0.50 ± 0.04 ^a^	0.23 ± 0.01 ^b^	**
cis-Linalool oxide	3.50 ± 0.29 ^a^	0.75 ± 0.06 ^f^	1.08 ± 0.12 ^e^	0.85 ± 0.08 ^f^	2.75 ± 0.28 ^c^	3.08 ± 0.32 ^b^	2.85 ± 0.25 ^c^	0.30 ± 0.02 ^g,h^	0.10 ± 0.00 ^h^	0.40 ± 0.02 ^g^	1.58 ± 0.13 ^d^	**
β-Linalool	2.10 ± 0.18 ^e^	2.10 ± 0.15 ^e^	2.68 ± 0.25 ^d^	10.83 ± 1.05 ^b^	2.15 ± 0.24 ^e^	2.73 ± 0.29 ^d^	10.88 ± 1.07 ^b^	2.73 ± 0.31 ^d^	10.88 ± 1.04 ^b^	11.43 ± 1.12 ^a^	5.85 ± 0.61 ^c^	**
α-Terpinolen	-	0.22 ± 0.00 ^c^	-	0.43 ± 0.05 ^b,c^	0.20 ± 0.01 ^c^	-	0.44 ± 0.03 ^b,c^	0.23 ± 0.01 ^c^	0.70 ± 0.05 ^a^	0.44 ± 0.02 ^b,c^	0.28 ± 0.01 ^c^	**
*trans*-Linalool oxide	1.53 ± 0.14 ^a^	0.40 ± 0.05 ^e^	0.58 ± 0.07 ^d,e^	0.45 ± 0.04 ^e^	1.13 ± 0.15 ^c^	1.30 ± 0.18 ^b,c^	1.20 ± 0.17 ^c^	0.18 ± 0.15 ^f,g^	0.08 ± 0.00 ^g^	0.23 ± 0.01 ^f^	0.70 ± 0.05 ^d^	**
Heptyl acetate	-	-	0.11 ± 0.00 ^a^	-	-	0.08 ± 0.00 ^a,b^	-	0.10 ± 0.01 ^a^	-	0.09 ± 0.00 ^a^	0.06 ± 0.00 ^b^	**
Neo-allo-ocimene	-	-	-	0.36 ± 0.03 ^a^	-	-	0.35 ± 0.02 ^a^	-	0.33 ± 0.03 ^a^	0.33 ± 0.02 ^a^	0.18 ± 0.01 ^b^	**
β-Citronellal	-	-	0.39 ± 0.04 ^a^	-	-	0.36 ± 0.03 ^a^	-	0.38 ± 0.04 ^a^	-	0.37 ± 0.05 ^a^	0.13 ± 0.00 ^b^	**
Nonanal	-	0.15 ± 0.00 ^e^	0.65 ± 0.08 ^c^	-	0.12 ± 0.00 ^e^	0.69 ± 0.08 ^b,c^	-	0.78 ± 0.06 ^a^	0.13 ± 0.00 ^e^	0.64 ± 0.05 ^c^	0.29 ± 0.01 ^d^	**
*trans*-3-Pinanone	-	-	0.03 ± 0.00 ^a^	-	-	0.03 ± 0.00 ^a^	-	0.02 ± 0.00 ^a^	-	0.01 ± 0.00 ^a^	0.01 ± 0.00 ^a^	**
1-Nonanol	-	-	0.63 ± 0.05 ^a^	-	-	0.58 ± 0.04 ^b^	-	0.61 ± 0.05 ^a,b^	-	0.59 ± 0.06 ^b^	0.27 ± 0.01 ^c^	**
4-Terpineol	0.68 ^f^	1.10 ± 0.15 ^c,d^	1.00 ± 0.12 ^d^	0.23 ± 0.19 ^g^	1.33 ± 0.21 ^b,c^	1.23 ± 0.23 ^c^	0.45 ± 0.05 ^f,g^	1.65 ± 0.22 ^a^	0.88 ± 0.09 ^e^	0.78 ± 0.07 ^e,f^	0.93 ± 0.09 ^d,e^	**
α-Terpineol	1.65 ^f^	2.05 ± 0.21 ^ef^	1.45 ± 0.15 ^g^	3.63 ± 0.34 ^c^	2.63 ± 0.25 ^d,e^	2.03 ± 0.19 ^e,f^	4.20 ± 0.34 ^a,b^	2.45 ± 0.23 ^e^	4.63 ± 0.38 ^a^	4.03 ± 0.31 ^b^	2.88 ± 0.29 ^d^	**
Decanal	-	-	0.45 ± 0.04 ^a^	-	-	0.46 ± 0.04 ^a^	-	0.45 ± 0.05 ^a^	-	0.40 ± 0.03 ^a,b^	0.24 ± 0.01 ^b^	**
cis-Geraniol	0.73 ^f^	1.10 ± 0.11 ^e^	1.48 ± 0.15 ^d^	2.73 ± 0.23 ^b^	1.00 ± 0.09 ^e^	1.38 ± 0.12 ^d,e^	2.63 ± 0.21 ^b^	1.73 ± 0.15 ^c,d^	2.98 ± 0.32 ^a,b^	3.38 ± 0.36 ^a^	1.90 ± 0.18 ^c^	**
β-Citral	1.05 ^f^	1.48 ± 0.15 ^d,e^	2.73 ± 0.28 ^a^	0.88 ± 0.08 ^f,g^	0.78 ± 0.06 ^g^	2.03 ± 0.16 ^c,d^	0.18 ± 0.01 ^i^	2.45 ± 0.23 ^b^	0.60 ± 0.03 ^h^	1.85 ± 0.12 ^d^	1.40 ± 0.11 ^e^	**
*trans*-Geraniol	0.58 ^f^	0.48 ± 0.02 ^g^	2.03 ± 0.21 ^d^	2.35 ± 0.24 ^c^	0.55 ± 0.04 ^f,g^	2.13 ± 0.18 ^c,d^	2.43 ± 0.22 ^b^	2.00 ± 0.19 ^d^	2.33 ± 0.23 ^bc^	3.88 ± 0.28 ^a^	1.88 ± 0.18 ^e^	**
1-Decanol	-	0.40 ± 0.03 ^a^	-	-	0.38 ± 0.02 ^a,b^	-	-	0.42 ± 0.03 ^a^	0.34 ± 0.02 ^b^	-	0.37 ± 0.04 ^a,b^	**
α-Citral	0.78 ^f^	1.30 ± 0.11 ^e^	5.03 ± 0.48 ^a^	0.78 ± 0.05 ^f^	0.53 ± 0.04 ^g^	4.25 ± 0.41 ^c^	-	4.78 ± 0.45 ^b^	0.53 ± 0.04 ^g^	4.25 ± 0.39 ^c^	2.23 ± 0.21 ^d^	**
Linalyl propionate	-	-	-	0.11 ± 0.01 ^a^	-	-	0.08 ± 0.00 ^a,b^	-	0.10 ± 0.00 ^a^	0.07 ± 0.00 ^a,b^	0.03 ± 0.00 ^b^	**
Neryl acetate	-	-	-	2.32 ± 0.25 ^a^	-	-	2.28 ± 0.22 ^a^	-	2.31 ± 0.24 ^a^	2.32 ± 0.23 ^a^	0.93 ± 0.08 ^b^	**
Geranyl acetate	-	-	-	2.34 ± 0.21 ^a^	-	-	2.35 ± 0.25 ^a^	-	2.31 ± 0.22 ^a^	2.36 ± 0.24 ^a^	0.99 ± 0.09 ^b^	**
Caryophyllene	0.18 ^e^	0.51 ± 0.04 ^b^	0.28 ± 0.02 ^d^	-	0.65 ± 0.06 ^a,b^	0.43 ± 0.04 ^c^	0.18 ± 0.01 ^e^	0.75 ± 0.08 ^a^	0.48 ± 0.05 ^bc^	0.28 ± 0.03 ^d^	0.38 ± 0.04 ^cd^	**
α-Bergamotene	-	-	0.39 ± 0.04 ^a^	-	-	0.36 ± 0.03 ^a^	-	0.36 ± 0.03 ^a^	-	0.38 ± 0.04 ^a^	0.17 ± 0.01 ^b^	**
β-Bisabolene	-	0.63 ± 0.07 ^a^	-	-	0.61 ± 0.06 ^a^	-	-	0.60 ± 0.05 ^a^	0.63 ± 0.06 ^a^	-	0.24 ± 0.02 ^b^	**
Epiglobulol	-	-	0.11^a^	-	-	0.11^a^	-	0.09 ^ab^	-	0.08 ^a,b^	0.04 ^b^	**
*trans*-Nerolidol	0.29 ± 0.03 ^a^	-	-	-	0.28 ± 0.03 ^a^	0.28 ± 0.02 ^a^	0.26 ± 0.02 ^a^	-	-	-	0.10 ± 0.00 ^b^	**
Nerolidyl acetate	-	-	0.07 ± 0.00 ^a^	-	-	0.06 ± 0.00 ^a^	-	0.03 ± 0.00 ^a^	-	0.03 ± 0.00 ^a^	0.02 ± 0.00 ^a^	**
*cis*-Farnesol	0.39 ± 0.02 ^ab^	0.44 ± 0.03 ^a^	0.48 ± 0.05 ^a^	0.46 ± 0.05 ^a^	-	-	-	-	-	-	0.18 ± 0.00 ^b^	**
Nootkatone	-	-	2.28 ± 0.23 ^a^	-	-	2.20 ± 0.21 ^a,b^	-	2.18 ± 0.22 ^b^	-	2.22 ± 0.23 ^a^	0.87 ± 0.09 ^c^	**
Monoterpene hydrocarbons	84.53	84.65	67.70	65.62	83.66	66.70	64.74	66.89	64.93	47.95	69.80	
Oxygenated monoterpenes	12.60	10.76	18.48	27.50	12.85	20.55	29.53	18.67	27.73	35.36	21.44	
Sesquiterpene hydrocarbons	0.18	1.14	0.67	-	1.26	0.79	0.18	1.71	1.11	0.66	0.79	
Oxygenated sesquiterpenes	0.68	0.44	2.94	0.46	0.28	2.65	0.26	2.22	-	2.33	1.21	
Others	-	0.55	4.10	-	0.50	4.02	-	4.68	0.47	4.04	2.16	

^ Sample obtained by combination P1, P2, P3, P4, and P5 EOs (1:1 *v*/*v*). ^a^ Linear retention index obtained for DB-5MS non-polar column; ^b^ linear retention index obtained for DB-Wax column; ^c^ content is the peak volume percentage of compounds in the essential oil sample; ^d^: 1 = retention index identical to bibliography; 2 = identification based on comparison of MS; 3 = retention time identical to authentic compounds. Compounds are classified in order of linear retention time of non-polar column. ^e^ Sign: Significance at ** *p* < 0.05. Results followed by different letters in the same line are significantly different (*p* < 0.05) by Tukey’s multiple-range test.

**Table 3:** Antioxidant activities of *C. maxima* cultivar EOs and their blends.

	ABTS (IC_50_ μg/mL)	DPPH (IC_50_ μg/mL)	FRAP μM Fe^2+^/g	β-Carotene Bleaching Test (IC_50_ μg/mL)
*C. maxima* EO				
P1	22.24 ± 2.07 ^a^	27.23 ± 2.02 ^b^	65.76 ± 3.27 ^g^	42.24 ± 2.66 ^j^
P2	31.82 ± 2.12 ^h^	35.32 ± 2.13 ^h^	58.59 ± 4.09 ^i^	32.22 ± 245 ^d^
P3	25.45 ± 1.66 ^c^	32.27 ± 2.64 ^g^	65.29 ± 3.12 ^g^	40.66 ± 2.80 ^i^
P4	32.56 ± 2.20 ^i^	35.26 ± 2.23 ^h^	64.67 ± 3.82 ^h^	25.56 ± 2.38 ^b^
P5	30.42 ± 2.45 ^g^	38.14 ± 2.92 ^i^	56.26 ± 3.63 ^j^	46.23 ± 2.73 ^l^
EO blends (1:1 v/v)				
P1P2	28.12 ± 2.23 ^e^	32.25 ± 1.81 ^g^	66.28 ± 3.12 ^f^	26.28 ± 2.11 ^c^
P1P3	29.01 ± 2.45 ^f^	35.12 ± 2.27 ^h^	69.56 ± 3.02 ^d^	21.87 ± 2.28 ^a^
P1P4	35.23 ± 2.12 ^l^	29.89 ± 2.78 ^d^	65.23 ± 3.11 ^k^	40.33 ± 2.87 ^i^
P1P5	31.24 ± 2.43 ^h^	31.71 ± 2.26 ^f^	69.12 ± 3.09 ^d^	35.23 ± 2.03 ^f^
P2P3	33.26 ± 2.56 ^j^	35.24 ± 2.95 ^h^	47.11 ± 3.04 ^l^	39.34 ± 3.02 ^h^
P2P4	31.12 ± 2.24 ^h^	28.76 ± 2.35 ^c^	68.89 ± 3.78 ^d,e^	34.22 ± 2.94 ^e^
P2P5	26.92 ± 2.45 ^d^	28.12 ± 2.31 ^c^	71.32 ± 3.09 ^c^	38.76 ± 2.07 ^g^
P3P4	32.31 ± 2.65 ^i^	32.25 ± 2.24 ^g^	72.33 ± 3.12 ^b^	32.72 ± 2.32 ^d^
P3P5	34.24 ± 2.78 ^k^	30.12 ± 2.20 ^e^	65.35 ± 3.20 ^g^	45.16 ± 2.94 ^k^
P4P5	35.27 ± 2.68 ^l^	29.22 ± 2.14 ^d^	72.78 ± 3.23 ^a^	48.51 ± 2.83 ^m^
Mix *^*	24.28 ± 2.71 ^b^	26.82 ± 2.98 ^a^	68.06 ± 3.54 ^e^	45.41 ± 3.34 ^k^
Sign.	**	**	**	**

^ Blend obtained by combination P1, P2, P3, P4, and P5 EOs (1:1 *v*/*v*). Data are expressed as means ± standard deviation (SD). The following positive controls were used: ascorbic acid in 2,2′-Azino-Bis-3-Ethylbenzothiazoline-6-Sulfonic acid (ABTS) (IC_50_ value of 1.72 ± 0.09 µg/mL) test and 2,2-Diphenyl-1-picrylhydrazyl (DPPH) (IC_50_ value of 5.03 ± 0.79 µg/mL); butylated hydroxytoluene (BHT) in ferric reducing ability power (FRAP) (FRAP value 63.27 ± 4.48 µM Fe(II)/g), and propyl gallate in β-carotene bleaching test (IC_50_ value of 0.09 ± 0.004 µg/mL); Sign: significance at ** *p* < 0.05. Results followed by different letters in the same line are significantly different according to Tukey’s multiple-range test.

**Table 4:** *C. maxima* EOs and their blends’ lipase-, α-amylase-, and α-glucosidase-inhibitory activities (IC_50_ μg/mL).

	Lipase	α-Amylase	α-Glucosidase
*C. maxima* EO			
P1	26.32 ± 2.32 ^d^	35.28 ± 2.13 ^j^	28.78 ± 2.24 ^f^
P2	34.12 ± 2.14 ^j^	34.22 ± 1.89 ^i^	25.67 ± 2.31 ^c^
P3	37.26 ± 2.53 ^l^	27.56 ± 2.82 ^e^	45.23 ± 2.21 ^k^
P4	23.22 ± 2.02 ^a^	25.23 ± 2.05 ^d^	48.69 ± 2.78 ^l^
P5	24.23 ± 2.12 ^b^	39.86 ± 2.54 ^m^	54.56 ± 3.45 ^m^
EO blends (1:1 v/v)			
P1P2	28.21 ± 2.12 ^e^	38.18 ± 2.21 ^l^	24.01 ± 1.88 ^b^
P1P3	33.87 ± 2.90 ^i^	36.19 ± 2.11 ^k^	27.87 ± 1.72 ^e^
P1P4	39.12 ± 2.61 ^m^	21.43 ± 2.05 ^a^	28.23 ± 2.02 ^f^
P1P5	30.95 ± 2.23 ^g^	22.46 ± 2.87 ^b^	37.26 ± 2.34 ^j^
P2P3	29.12 ± 2.45 ^f^	30.22 ± 2.18 ^g^	29.12 ± 2.62 ^g^
P2P4	25.22 ± 2.46 ^c^	24.71 ± 2.19 ^c^	26.55 ± 2.76 ^d^
P2P5	26.76 ± 2.69 ^d^	28.55 ± 2.23 ^f^	21.67 ± 1.98 ^a^
P3P4	28.24 ± 2.08 ^e^	31.05 ± 2.94 ^h^	32.05 ± 2.03 ^h^
P3P5	35.06 ± 2.12 ^k^	36.45 ± 2.98 ^k^	27.85 ± 2.28 ^e^
P4P5	31.12 ± 2.05 ^h^	30.23 ± 2.75 ^g^	34.67 ± 2.65 ^i^
Mix ^	30.12 ± 2.52 ^g^	25.67 ± 2.05 ^d^	28.12 ± 2.50 ^f^
Sign.	**	**	**

^ Blend obtained by combination P1, P2, P3, P4, and P5 EOs (1:1 *v*/*v*). Data are expressed as means ± standard deviation (SD). The following positive controls were used: Acarbose for α-amylase (IC_50_ value of 35.53 ± 1.28 μg/mL) and α-glucosidase (IC_50_ value of 50.09 ± 1.34 μg/mL); Orlistat for lipase (IC_50_ value of 37.42 ± 1.08 μg/mL). Sign: significance at ** *p* < 0.05. Results followed by different letters in the same line are significantly different according to Tukey’s multiple-range test.

## Data Availability

All data and materials are available upon request from the corresponding author.

## Supplementary Materials

The following supporting information can be downloaded at: https://www.mdpi.com/article/10.3390/molecules27103273/s1. Figure S1: Chromatogram obtained by injection of C. maxima ‘Chadock’ (P1) EO; Figure S2: Chromatogram obtained by injection of C. maxima ‘Maxima’ (P2) EO; Figure S3: Chromatogram obtained by injection of C. maxima ‘Pyriformis’ (P3) EO; Figure S4: Chromatogram obtained by injection of C. maxima ‘Terracciani’ (P4) EO; Figure S5: Chromatogram obtained by injection of C. maxima ‘Todarii’ (P5) EO.

## Sample Availability

Samples of *C. maxima* essential oils are available from the authors.
