# Supplementary material for: Reuse of Food Waste: The Chemical Composition and Health Properties of Pomelo (Citrus maxima) Cultivar Essential Oils
Source: Molecules. 2022 May 19;27(10):3273. doi: 10.3390/molecules27103273 (PMC9146573; doi:10.3390/molecules27103273)
Supplement: Supplementary file 1 [file molecules-27-03273-s001.zip › molecules-1735806-supplementary.pdf]

Supplementary material

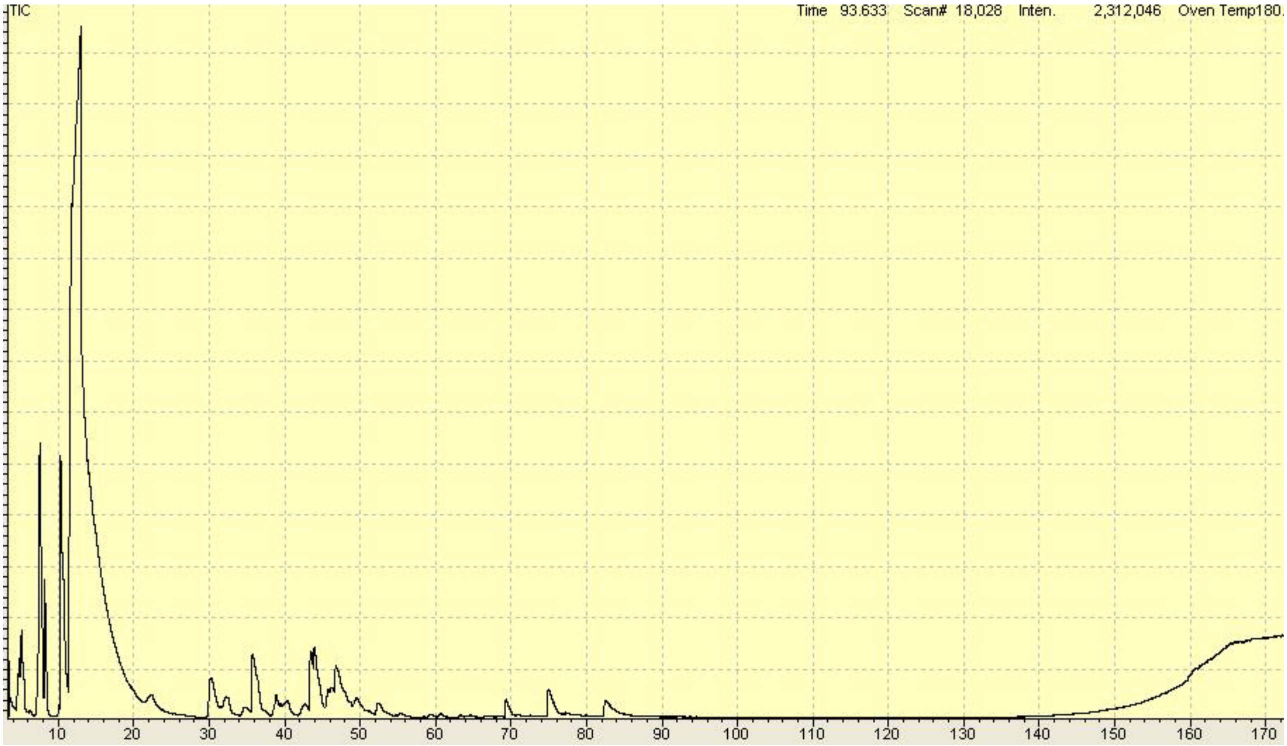

Figure S1: Chromatogram obtained by injection of *C. maxima* 'Chadock' **(P1)** EO.

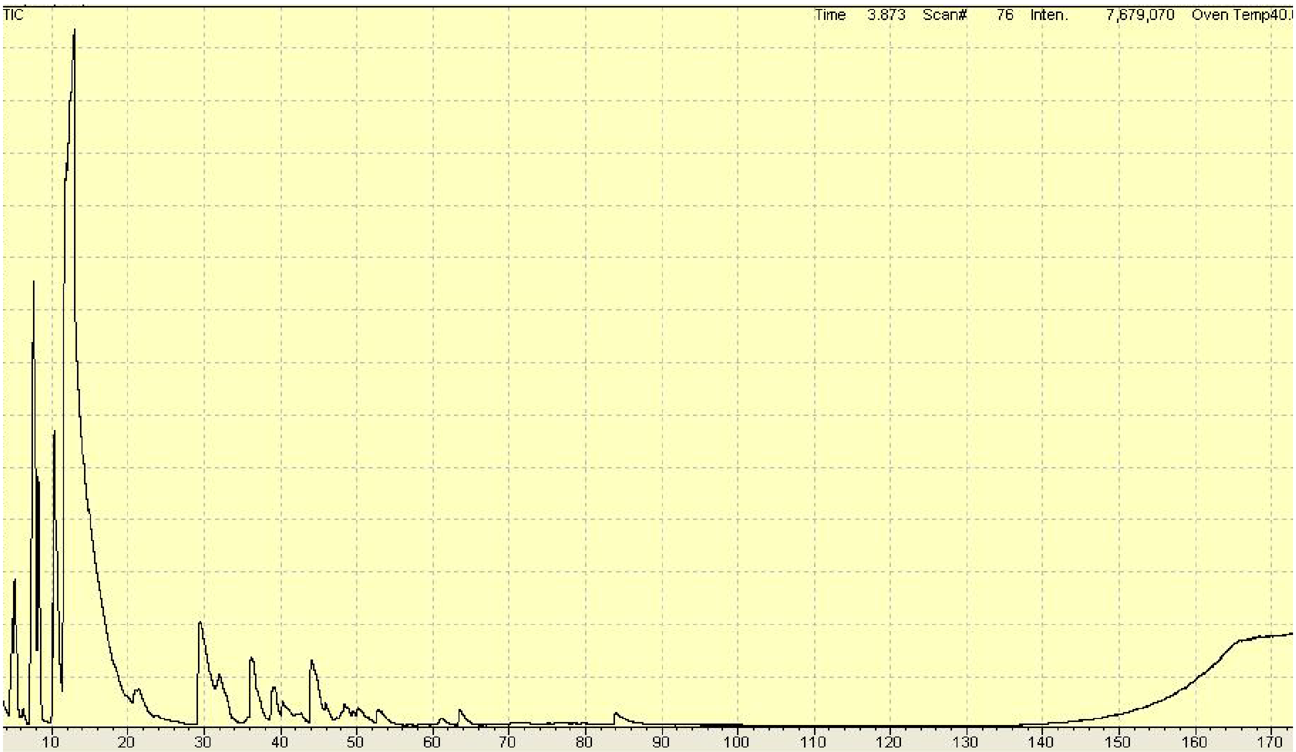

Figure S2. Chromatogram obtained by injection of *C. maxima* 'Maxima' (**P2**) EO.

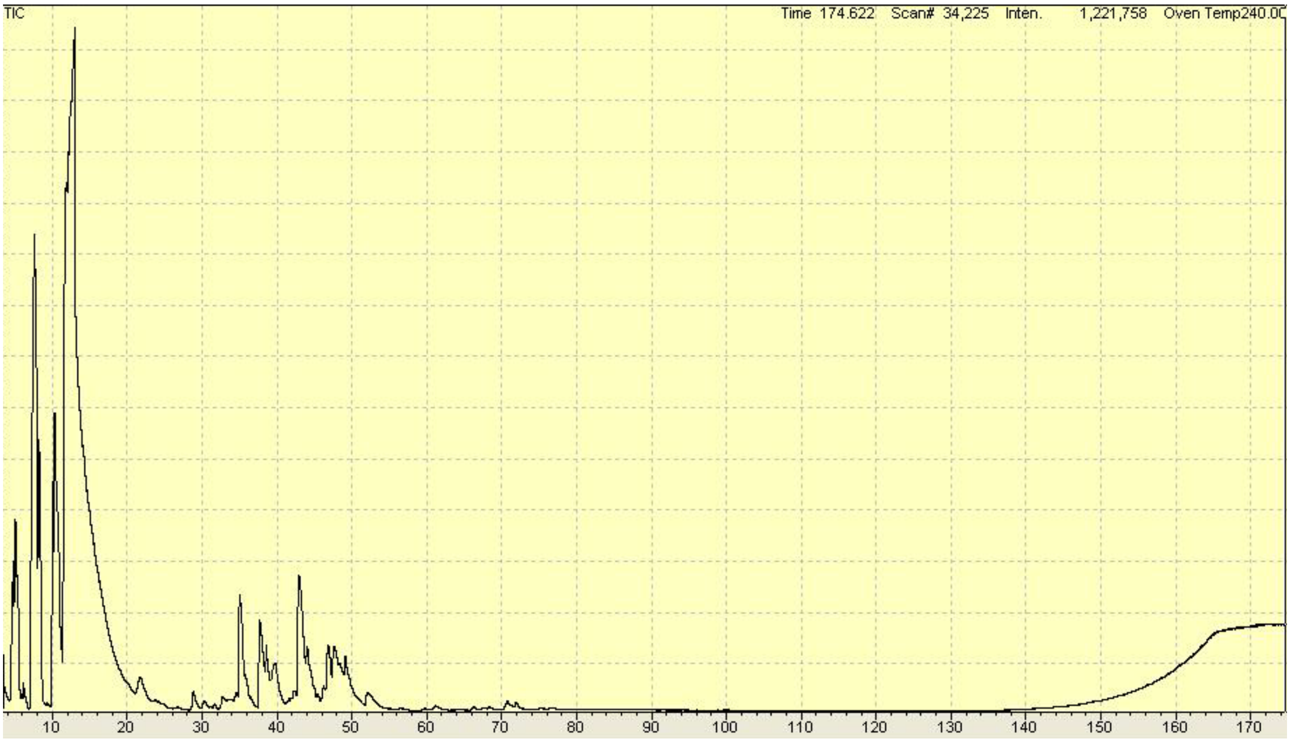

Figure S3. Chromatogram obtained by injection of *C. maxima* ‘Pyriformis’ (**P3**) EO.

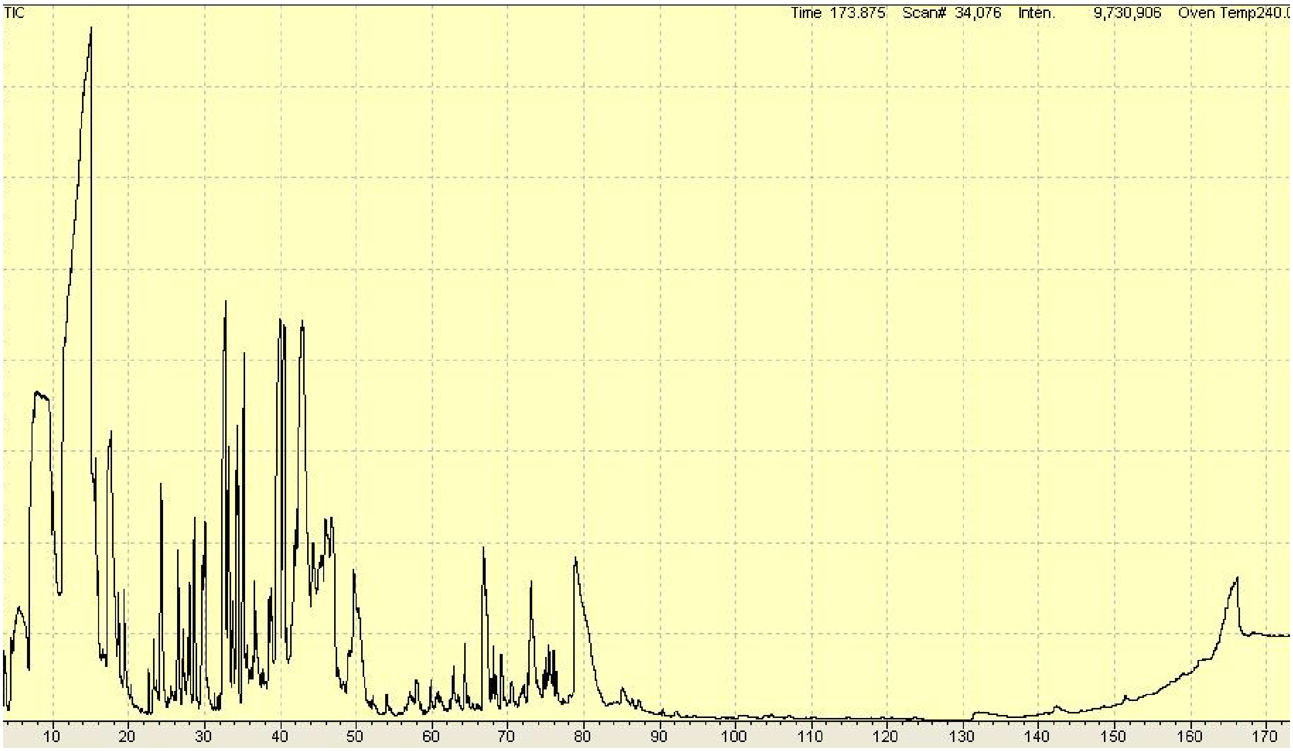

Figure S4. Chromatogram obtained by injection of *C. maxima* ‘Terracciani’ (**P4**) EO.

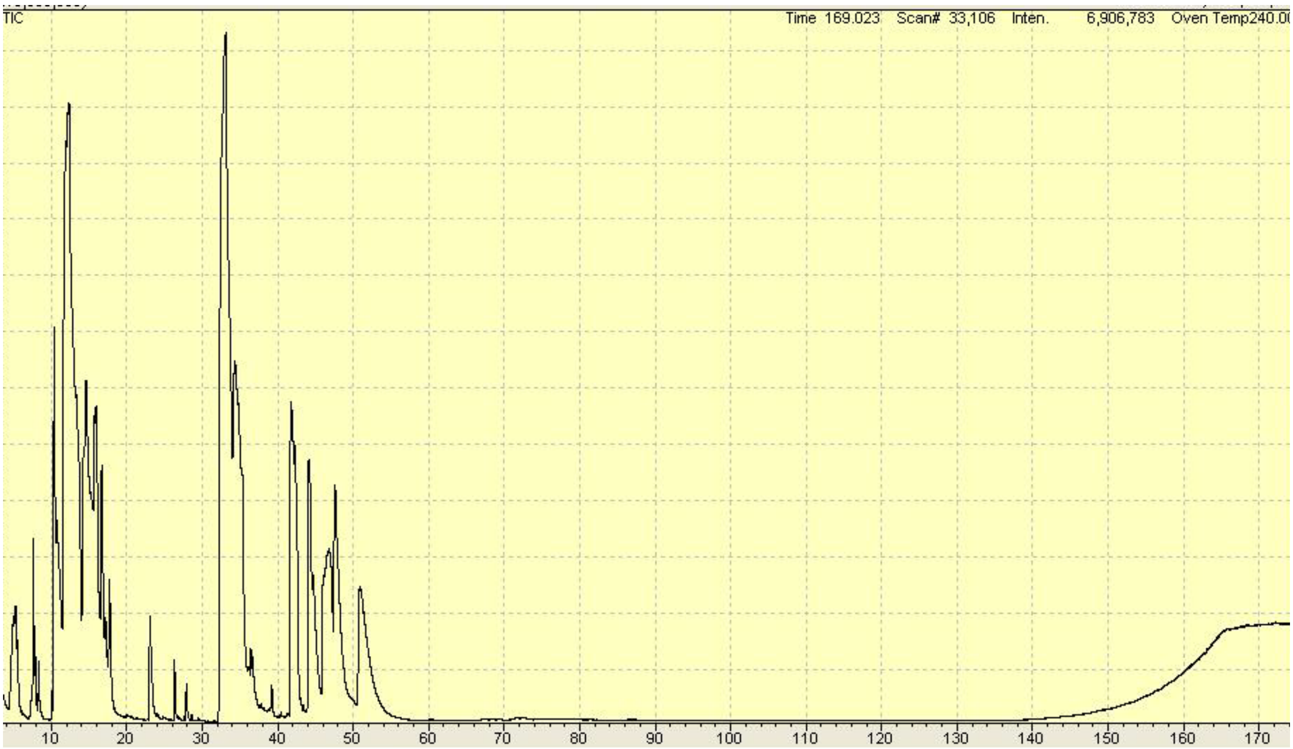

Figure S5. Chromatogram obtained by injection of *C. maxima* 'Todarii' (P5) EO.
